# Geospatial impact evaluation of a low-cost agricultural intervention for enhancing environmental resilience^☆^

**DOI:** 10.1016/j.jag.2025.104657

**Published:** 2025-07

**Authors:** Pratap Khattri, Rachel Sayers, Kunwar K. Singh, Ryan Slapikas, Chet Bahadur Tamang, Dinee Tamang, Brad Sagara, Ariel BenYishay

**Affiliations:** a AidData, Global Research Institute, William & Mary, Williamsburg, VA 23185, USA; b Center for Geospatial Analysis, William & Mary, Williamsburg, VA 23185, USA; c Department of Geography, Florida State University, Tallahassee, FL 32306, USA; d Managing Risk Through Economic Development, Mercy Corps, Lalitpur 44700, Nepal; e Research & Learning, Mercy Corps, Portland, OR 97204, USA; f Department of Economics, William & Mary, Williamsburg, VA 23185, USA

**Keywords:** Geospatial impact evaluation (GIE), Developmental interventions, Impact evaluation, Sugarcane farming, Riverbank degradation, Difference-in-difference (DID), Machine-learning algorithms

## Abstract

Land degradation poses a significant threat to ecosystems and livelihoods, particularly in disaster-prone regions. In these settings, the promotion of certain agricultural practices with economic incentives, such as sugarcane (*Saccharum officinarum*) farming, offers a potential solution for enhancing economic stability and mitigating environmental degradation. Despite its promise, the effectiveness of sugarcane as an agricultural intervention remains understudied, especially regarding its environmental benefits. Our study evaluates the impact of sugarcane cultivation in western Nepal, a region highly vulnerable to soil erosion and riverbank degradation due to the presence of flood-prone landscapes. We conducted a geospatial impact evaluation (GIE), which integrated remote sensing data and econometric techniques, including optimal full matching (OFM) and difference-in-differences (DID). We assessed the causal impact of a program promoting sugarcane farming on its adoption and environmental outcomes, measured using multi-temporal satellite imagery, crop phenology, and clustering algorithms, along with machine learning and visual interpretation methods. Our results show that sugarcane adoption increased significantly in both treated and spillover communities, highlighting its potential as a sustainable agricultural practice. However, while uptake was evident, the expected environmental outcomes, such as soil erosion control and riverbank stabilization, did not materialize. This study demonstrates the potential of GIE in evaluating low-cost interventions for sustainable development and provides insights into the role of sugarcane cultivation in promoting climate resilience. The findings underscore the need for complementary interventions and extended timeframes to realize long-term environmental benefits, contributing valuable evidence for policymakers and development practitioners.

## Highlights

• Geospatial impact evaluation optimizes development program evaluation and outcomes.
• Random forest algorithm & crop phenology aided annual sugarcane distribution mapping.
• Satellite image interpretation quantifies river boundaries and erosion areas.
• OFM and DID identify program effects when lacking a pre-selected comparison group.
• Low-cost sugarcane adoption with economic incentives can drive self-sustaining growth.

## 1 Introduction

Low-cost agricultural interventions, such as alternate wetting and drying (Alauddin et al., 2020), drip irrigation (Del Carpio et al., 2011), and deep-rooted crops (De Baets et al., 2007), offer viable solutions to enhance economic stability and climate resilience while mitigating environmental degradation in resource-limited communities (Piñeiro et al., 2020). Among these, sugarcane (*Saccharum officinarum*) cultivation has shown potential to reduce soil erosion, stabilize flood-prone landscapes, and improve livelihood (Cheng et al., 2022). However, most assessments of sugarcane’s impacts rely either on remote sensing or econometric methods, rarely integrating both to establish causal relationships. Traditional impact evaluation (IE) approaches using ground-based data help isolate treatment effects but are often constrained by the lack of pre-identified comparison groups, a cornerstone of causal inference, limiting their utility in real-world contexts (White and Raitzer, 2017). Geospatial Impact Evaluation (GIE) presents a compelling alternative by integrating remote sensing data with IE methods to generate spatially relevant comparison groups and enable causal analysis (BenYishay et al., 2017, Singh et al., 2024). Despite its potential, GIE has rarely been applied to evaluate the impacts of sugarcane cultivation as a low-cost agricultural intervention (Prove et al., 1995), leaving it unclear whether such cultivation can both mitigate environmental degradation and providing economic benefits, or if alternative strategies might be more effective.

Bioengineering measures, such as cultivating deep-rooted crops, can effectively reduce erosion and stabilize riverbanks across diverse settings (Mallik and Rasid, 1993, Abernethy and Rutherfurd, 1998, Abernethy and Rutherfurd, 2000). Vegetation strengthens soil structure and microclimate conditions that enhance erosion control (Boer and Puigdefábregas, 2005, Vásquez-Méndez et al., 2010). However, adoption has been limited due to socioeconomic constraints, lack of technical support, and inadequate access to resources (Barnes et al., 2019, Caviglia-Harris, 2003, Garbach et al., 2012, Teklewold et al., 2013). Targeted support, such as training programs, subsidies, and economic incentives, can improve uptake by aligning environmental goals with economic incentives (Piñeiro et al., 2020). These innovations often diffuse through farming networks, producing spillover effects that contribute to broader climate resilience (Klerkx et al., 2012). Mercy Corps’ Managing Risk through Economic Development (M−RED) program in western Nepal bundled sugarcane cultivation with market development, training, and technical assistance to connect farmers to local sugar mills. While the program has gained traction within communities, its broader environmental and economic outcomes remain unclear, highlighting the need to assess its long-term viability and potential trade-offs.

Sugarcane’s dense roots and foliage can help stabilize riverbanks by improving soil retention and reducing runoff (Cardoso et al., 2018, El Chami et al., 2020). However, its effectiveness as an erosion control strategy remains underexplored. Reported erosion outcomes vary widely—from 31 tons/hectare/year in Brazil to 148 in Australia—due to differences in harvesting practices, land-use history, and climate (Prove et al., 1995, Sparovek and Schnug, 2001). Existing research is geographically limited and typically focuses on small river segments or controlled experiments (Cheng et al., 2022, Gholami and Khaleghi, 2013, Zhu et al., 2018). Most studies rely on correlation analysis rather than causal methods (Hu et al., 2021) and often lack control groups, increasing the risk of confounding effects (Cheng et al., 2022). Spillover effects are rarely considered, making it difficult to assess the long-term effectiveness of interventions (Piñeiro et al., 2020). To fill these gaps, integrated approaches using remote sensing data and IE techniques are needed to generate more robust and scalable evidence across diverse ecological and socioeconomic settings.

Most studies rely on remote sensing-based land-use classification and elevation data at field or farm scales to assess vegetation’s effect on soil erosion and riverbank stabilization (Cheng et al., 2022, Zhu et al., 2018), limiting their ability to capture broader landscape dynamics (Mondal & Patel, 2020). These analyses often use static or cross-sectional spatial data, overlooking temporal changes in land-use, climate, and plantation management that shape erosion outcomes. Such limitations arise from the inability to extend beyond ground observations, especially in hard-to-survey areas, and from the lack of long-term time-series data to establish pre-intervention baselines (e.g., land use, forest cover). High-resolution, multi-spectral satellite imagery, such as Sentinel-2 (10–20 m) and PlanetScope (3–5 m), offers improved spatial and temporal resolution for delineating sugarcane fields. When paired with ground observation, these data enable more accurate mapping of sugarcane farms (Som-Ard et al., 2021, Adami et al., 2012). Machine learning techniques—including random forests and convolutional neural networks—further enhance land-use classification and erosion monitoring (Wang et al., 2019), while visual interpretation can aid in detecting riverbank erosion and soil degradation (Fadul et al., 1999;). Yet few studies use long-term time-series data to establish pre-intervention baselines or apply quasi-experimental methods, such as difference-in-differences (DID) and matching techniques, to estimate causal impacts. Integrating high-resolution remote sensing with causal inference is essential to assess the effectiveness, scalability, and resilience of sugarcane interventions across diverse ecological and climatic settings (BenYishay et al., 2017, Singh et al., 2024). These methods have been used to assess the impacts of small-scale irrigation on crop productivity (BenYishay et al., 2024), winds on wheat yields (Wang et al., 2021), stripe rust on crop reflectance (Jing et al., 2022), and mining-induced ecological changes (Liu et al., 2023). DID reduces selection bias by ensuring comparability between treated and untreated areas (Angrist & Pischke, 2009), while matching approaches improve comparability by balancing geographic, soil, and socioeconomic characteristics (Zhang et al., 2023). Therefore, integration of geospatial and causal inference methods enhances both the scalability and effectiveness of sugarcane interventions.

In this study, we conduct a GIE to assess the effectiveness of sugarcane cultivation in reducing soil erosion and mitigating riverbank degradation in disaster-prone communities of western Nepal. Using Mercy Corps’ long-standing M−RED program, we integrate multi-temporal remotely sensed satellite imagery with geospatial analytics, such as crop phenology analysis, clustering algorithms, and visual interpretation, to map sugarcane farms and quantify changes in environmental conditions. By generating a valid comparison group through optimal full matching (OFM) and then combining this with DID, a robust causal inference method, we control for confounding factors and selection bias, ensuring more unbiased estimations of the causal impacts of sugarcane adoption. We then assess spillover effects on neighboring communities by evaluating changes in sugarcane adoption rates and environmental outcomes. By examining how sugarcane adoption in treated areas influences surrounding regions, this study provides a comprehensive understanding of sugarcane’s broader appeal and its potential as both a sustainable income source and a practice for mitigating riverbank degradation.

## 2 Material and methods

### 2.1 Study area

**Fig. 1:**
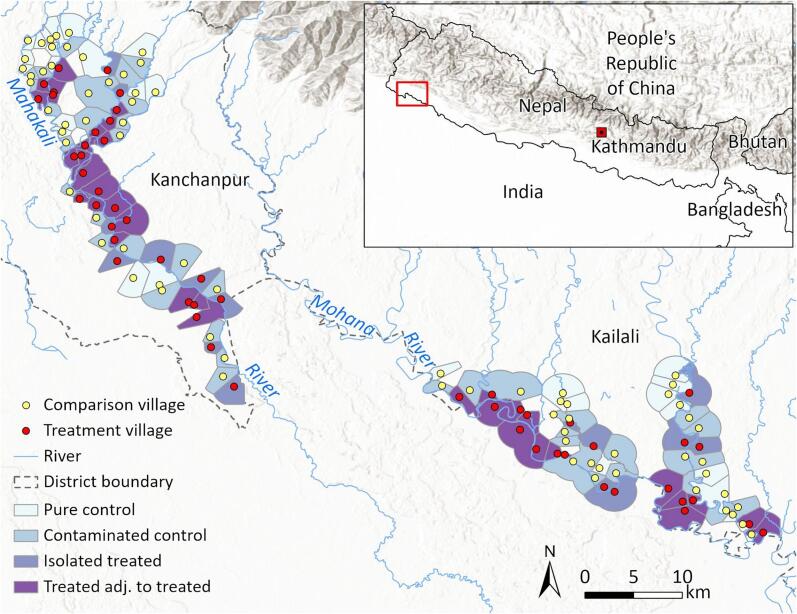
Study area in Nepal’s far-western Terai region, highlighting treatment (red) and comparison villages (yellow) with their corresponding buffers, classified by proximity to treated areas. Buffers are shown relative to the Mahakali and Mohana rivers (blue). Inset map highlights Kanchanpur and Kailali districts, focus of the Managing Risks through Economic Development (M-RED) program.

The study focuses on communities in the Kailali and Kanchanpur districts of Nepal’s western Terai region to assess the potential of sugarcane cultivation as a cost-effective intervention for reducing soil erosion and riverbank degradation (Fig. 1). Located along the northern edge of the Indo-Gangetic Plain, the Terai is divided into three geomorphic zones: Bhabar, Middle Terai, and Lower Terai (Ghimire & Basnet, 2015). The terrain consists of Quaternary alluvial deposits and flat land shaped by shifting river channels. Although agriculturally productive, the region is highly vulnerable to monsoonal flooding, worsened by human activity and climate change. Seasonal floods regularly damage crops, erode soil, and destroy infrastructure (Sharma et al., 2013). From 2010 to 2021, floods affected 27 % of the population in Kailali and Kanchanpur and submerged up to 40 % of farmland during peak events, causing annual losses averaging 1.2 billion Nepalese rupees (Bhandari, 2021). The landscape is predominantly agricultural, interspersed with tropical and subtropical vegetation, including Sal (*Shorea robusta*) forests, grasslands, and wetlands. Agriculture is the primary land use, with key crops including rice, maize, sugarcane, sesame, and vegetables. Since the M−RED program began, sugarcane cultivation has expanded significantly, exceeding 8,000 ha in Kanchanpur by 2020 and producing nearly 400,000 metric tons (Pathak, 2023). Irrigation is primarily from rainfall and river water, with increasing use of electric pumps for groundwater.

### 2.2 Development intervention

With support from Margaret A. Cargill Philanthropies, Mercy Corps implemented the M−RED program in the Terai region by integrating disaster risk reduction (DRR) with economic incentives. In collaboration with local communities, the program introduced sugarcane planting along erosion-prone riverbanks, converting barren land into productive areas. This approach reduces flood risk and soil erosion while providing farmers with income through sugarcane sales to local mills.

**Fig. 2:**
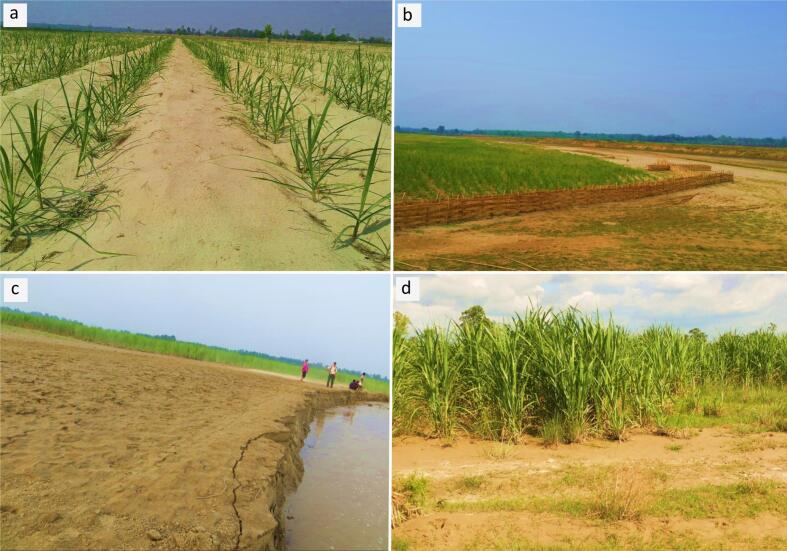
Sugarcane planted along sandy riverbanks helps stabilize erosion-prone soil (a). Bamboo reinforcements protect young sugarcane from river cutting, improving crop survival (b). Strategically planted and reinforced areas demonstrate effective protection against erosion (c). A sugarcane field following severe flooding illustrates the crop’s resilience to extreme weather and highlights ongoing challenges in managing flood-related damage (d).

Unlike traditional DRR approaches such as retaining walls—which are costly and often ineffective in the long-term—sugarcane offers a natural, low-maintenance alternative that can significantly reduce flooding impacts and prevent riverbank erosion (Chaulagain and Rimal, 2019, Neupane et al., 2019). Its deep roots and dense canopy stabilize the soil and reduce runoff, while its flood tolerance and profitability enhance farmers' resilience to climate-related risks (Dhakal et al., 2016, Neupane et al., 2017, Pokharel et al., 2019). The sandy soils of the Terai are particularly well-suited for sugarcane cultivation (Neupane et al., 2017), which thrives under prolonged flooding without yield loss (Glaz and Morris, 2010). Once established, it does not require tillage, further preventing erosion. The dense canopy of sugarcane reduces the direct impact of rainfall on the soil, while its interconnected root system improves soil cohesion, thereby minimizing surface runoff and soil erosion (Fig. 2). Collectively, these characteristics make sugarcane a highly effective and sustainable measure for controlling erosion in flood-prone areas.

### 2.3 M−RED program implementation

The M−RED program, implemented in Nepal’s Kailali and Kanchanpur districts, promoted sugarcane cultivation alongside market development to enhance agricultural resilience and economic stability. The intervention combined technical training in sugarcane farming with strategies to connect farmers to local sugar mills, while also supporting access to financial services and training in sustainable land management.

Communities for M−RED activities were selected through consultations with government and non-government stakeholders at both district and local levels. A scoring system, based on community-completed questionnaires, assessed disaster risk and economic development potential. Communities with high disaster risk and moderate to high economic opportunity were chosen for participation. Priority was also given to those previously supported by Mercy Corps through the SAFER-Nepal (Strengthening Actions for Fostering Resilience through Early Warning and Risk Sensitive Planning in Nepal) project.

The program was phased across 63 communities, grouped into five cohorts by year of completion. For evaluation, the analysis focuses on 53 communities where intervention zones intersect with riverbanks—critical for assessing erosion-related outcomes (Table 1). The first cohort (M−RED−1) was excluded due to insufficient pre-2016 sugarcane adoption data, which precluded baseline comparisons. This phased, targeted design supports a rigorous assessment of M−RED’s integrated resilience-building approach.

**Table 1:** The distribution of villages across treatment phases in the Managing Risks through Economic Development (M-RED) intervention illustrates the program’s staggered rollout, with new villages added in each phase to enable evaluation of its causal impacts over time.

M-RED phase	Completion year	Villages
M-RED 1*	2016	17
M-RED 2	2019	9
M-RED 2 Cost Extension	2020	8
M-RED 3	2021	11
M-RED 4	2022	8

* The phase was excluded from the analysis sample due to the absence of pre-treatment data on sugarcane adoption.

### 2.4 Village sample construction and matching process

We focused on two river systems in western Nepal to evaluate the impact of the M−RED program. To identify relevant villages, we combined two spatial datasets: a layer of treated villages provided by Mercy Corps and a comprehensive settlement layer from the UN Office for the Coordination of Humanitarian Affairs (OCHA, 2015; https://www.unocha.org). For each river, we selected all villages from the OCHA layer that were within 5 km of the river centerline, identifying villages likely to be influenced by riverine conditions. We then matched village names and administrative boundaries to align treated villages with corresponding OCHA entries.

We followed a multi-step process to create a pool of suitable comparison villages. First, we used the OCHA settlement layer and removed all villages identified as treated under the M−RED program. To account for possible mismatches in village names between the OCHA data and M−RED records, we also excluded any settlements located within a 1 km radius of treated villages. To further refine our sample to villages closely situated along river systems, we calculated the farthest distance of any treated village from the river for each system—2.1 km for the first and 1.5 km for the second—and excluded any potential comparison village beyond these thresholds. This resulted in a geographically comparable set of untreated villages from the OCHA layer and a corresponding set of treated villages based on project data.

We generated Thiessen (Voronoi) polygons around the sample villages to create distinct buffer zones for each (Fig. 1). We removed a 2-meter-wide buffer around the 2016 river centerline from the Thiessen polygons and conducted further cleaning to ensure that the village buffer regions did not extend across the river. We restricted the sample to villages with at least one buffer edge touching the river, as the sugarcane planting promoted by M−RED targeted riverbanks. Villages without such a buffer edge were excluded, and the buffers remained constant throughout the study period in our analysis. Finally, we excluded potential comparison villages whose buffer touched the buffer of any treated village, which we refer to as “contaminated comparisons”, and these villages comprise the treated group in our spillover sample. This restriction was implemented for two reasons: first, to avoid misattributing land managed by treated villages to comparison villages; and second, to reduce the influence of geographical spillovers, as non-treated villages close to treated villages had expressed interest in adopting sugarcane cultivation. Dropping these villages yielded the actual comparison group used in our analysis, which we refer to as “pure comparisons”.

### 2.5 Performing geospatial impact evaluation

**Fig. 3:**
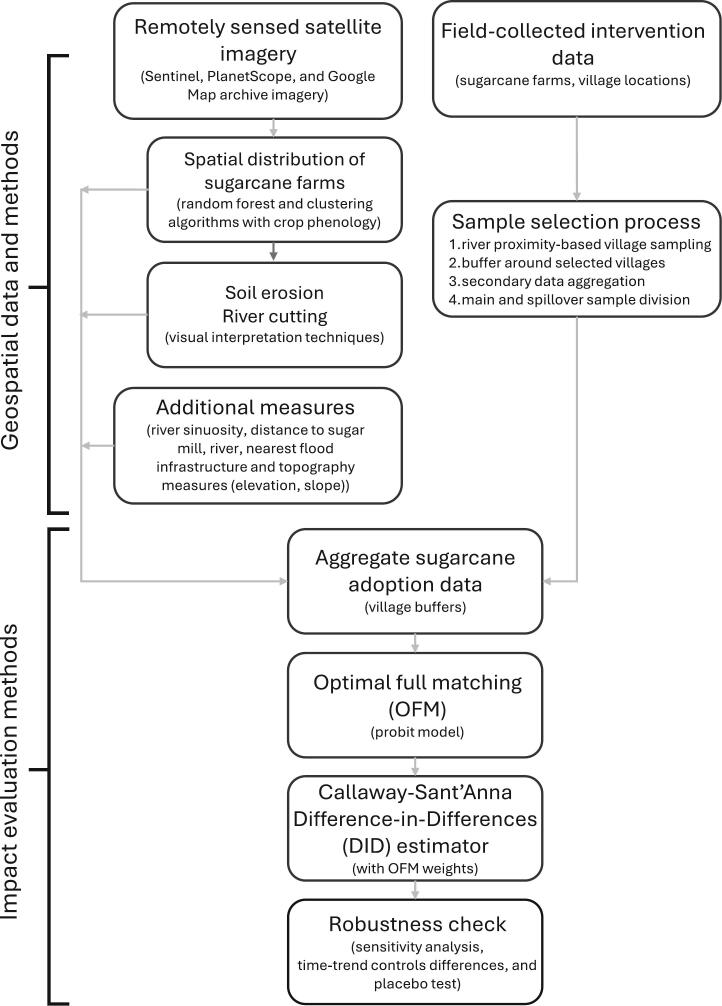
Schematic of the analytical framework, highlighting the methodological flow from data collection to final analysis.

To evaluate the M−RED program using a GIE framework, we combined satellite imagery with a DID approach to assess environmental outcomes (Singh et al., 2024). The process was executed in several structured steps (Fig. 3). Using post-monsoon Sentinel-2 imagery with < 30 % cloud cover, we mapped sugarcane cultivation across western Nepal. We applied hierarchical clustering and Random Forest classification, using spectral bands (red, blue, green, NIR) and vegetation indices such as the normalized difference vegetation index (NDVI). Sentinel-2 imagery, with 10-meter resolution, offered sufficient spectral, spatial, and temporal detail to reliably differentiate sugarcane from other crops and land cover types (Hunt et al., 2019).

We used a Random Forest algorithm to detect soil erosion and river shifts along riverbanks using post-monsoon imagery. However, detection accuracy was limited due to the small, fragmented nature of these features. To improve precision, we supplemented this with visual interpretation of high-resolution PlanetScope imagery, which provides daily 3-meter resolution data from CubeSats like Doves and SuperDoves (Planet, 2021; https://www.planet.com). We used orthorectified top-of-atmosphere and surface reflectance products, suitable for both machine learning and visual analysis. When PlanetScope imagery was unavailable for certain years, we relied on high-resolution Google Earth imagery. This multi-source approach enhanced detection of fine-scale erosion and river dynamics (Hu et al., 2013).

Finally, to construct the matched sample for our DID analysis, we incorporated variables derived from satellite imagery and proprietary data provided by Mercy Corps. The matching model included 12 selected variables (Table 2) to account for factors influencing both treatment probability and outcome trajectories across the sampled villages (Caliendo and Kopeinig, 2008). This approach enabled a robust, multi-scale assessment of how sugarcane cultivation influenced soil erosion and river system dynamics.

**Table 2:** Variables used in the probit model to estimate each village’s propensity score, selected for their relevance to treatment assignment and outcome trajectories.

Variables	Description
Distance to sugar mill	Euclidean distance to nearest sugar mill from the village center (meters)
Distance to river	Minimum Euclidean distance to the river from the village center (meters)
Distance to nearest flood infrastructure	Euclidean distance to the nearest flood mitigation infrastructure
Flood risk level	Village buffer-level flood risk
Population count	Village buffer-level population count in 2015
Built-up area	Village buffer-level built-up area in 2015 (square meters)
Mean slope	Average slope within 100 meters of the river section adjacent to a village buffer
Mean elevation	Average elevation within 100 meters of the river section adjacent to a village buffer (meters)
Average baseline sinuosity	Average sinuosity of the river section adjacent to a village buffer in 2016
Baseline pct. of land planted with Sugarcane	Percentage of village buffer within 250 meters from the river that is covered by sugarcane in 2016
Baseline pct. of land affected by soil erosion	Percentage of village buffer within 250 meters from the river that experienced soil erosion in 2016
Baseline average distance to near boundary	Average distance of the village center from the near river boundary in 2016 (meters)
Location of village	Location of village w.r.t the river systems.

#### 2.5.1 Mapping sugarcane acreage

To evaluate the effects of sugarcane cultivation on soil erosion, river cutting, and potential spillovers, we mapped annual sugarcane coverage alongside land cover classes such as forest, water, bare soil, sand, and other crops to improve classification accuracy. Given the diversity of cropping systems in the study area, we tested four vegetation indices (Vis): NDVI, soil adjusted vegetation index (SAVI), red edge position index (REP), and enhanced vegetation index (EVI). NDVI is a common indicator of vegetation health but can experience saturation issues, particularly in areas with exposed soil (Cao et al. 2014). SAVI addresses this by minimizing soil background interference (Rondeaux et al., 1996), while REP and EVI further reduce saturation, improving vegetation detection in heterogeneous landscapes (Gholizadeh et al., 2016, Huete et al., 2002).

For crop classification, we employed both hierarchical clustering and Random Forest algorithms. Ground reference data from 2015 were combined with crop phenology profiles through clustering techniques to map crop types. Hierarchical clustering, an unsupervised method, grouped samples based on similarity in time-series data using the nearest neighbor approach (Xu and Wunsch, 2008), making it particularly useful when *in-situ* data were unavailable. The hierarchical clustering grouped similar features into smaller clusters and dissimilar ones into larger clusters, which are then represented in a dendrogram (Gonçalves et al., 2008). We employed the 'ward.D' method in R's ‘hclust’ function, which minimizes within-cluster variance (Ward et al., 1963). To accomplish this, we used time-series NDVI data from Sentinel-2 imagery to create phenological profiles for each farm by averaging pixel values within farm boundaries across acquisition dates for each vegetation index. These profiles formed phenology curves representing crop growth cycles. To reduce noise and align the data with expected crop patterns, we applied a Savitzky-Golay filter, which smoothed time-series data through a weighted moving average (Dai et al., 2017). This approach capitalized on crop phenology dynamics to accurately detect crop types at the farm level while clustering similar entities (Hu et al., 2021). In parallel, we applied the supervised Random Forest algorithm (Breiman, 2001), using 40 decision trees and an 80/20 train-test split with default parameters. Known for its robustness to noise and resistance to overfitting (Belgiu and Drăguţ, 2016, Chan et al., 2012), Random Forest aggregated multiple decision trees built from random subsets of the data.

To assess classification performance, we used overall accuracy, Kappa, and the Matthews Correlation Coefficient (MCC), with MCC offering a more balanced evaluation due to Kappa’s sensitivity to chance agreement. Accuracy ranged from 87 % to 93 %. We also calculated user and producer accuracy, entropy, purity, and quantity/allocation disagreement for a comprehensive evaluation. Feature importance analysis from the Random Forest model highlighted key variables driving crop classification.

#### 2.5.2 Mapping soil erosion patterns through visual interpretation and spatial analysis

We mapped annual soil erosion using visual interpretation of high-resolution post-monsoon satellite imagery and aerial photos. Key erosion features—such as gullies, rills, bare soil, and sediment deposits—were identified based on their spectral, textural, and spatial characteristics (Zhang et al., 2012). Imagery from Google Earth (Taylor and Lovell, 2012) and PlanetScope imagery supported this process, with additional refinement through ancillary data and expert input from local professionals. While less scalable than automated methods, manual interpretation offers higher accuracy in complex landscapes, especially where training data for machine learning models are unavailable (Domlija et al., 2019). It is particularly effective for detecting subtle patterns often missed by automated classification. Expert knowledge from local professionals in Nepal further enhanced interpretation accuracy. We digitized exposed soil layers from 2016 to 2023 and quantified erosion by calculating the total area of exposed soil within each village buffer and within 250 m of the river centerline. These spatially defined zones allowed for targeted assessment of erosion near vulnerable riverbanks. By aggregating exposed soil area over time, we constructed a variable reflecting year-to-year changes in soil exposure, serving as a key metric to evaluate the impact of sugarcane adoption on soil erosion in the study region.

#### 2.5.3 Digitizing river boundaries for assessing cutting and shifting

Due to the absence of ground observation data, we used visual interpretation to map annual river boundaries and assess river cutting. High-resolution satellite imagery from Google Earth and PlanetScope was used to manually digitize river edges post-monsoon from 2016 to 2023. Expert interpreters in Nepal analyzed spectral signatures, color contrast, and geomorphological features to distinguish water bodies from adjacent land. These boundaries were digitized in a Geographic Information System environment using fine-scale vector lines that captured the river’s natural curves, meanders, and oxbows. Digitized layers were stored in a spatial database to enable temporal analysis of river changes.

**Fig. 4:**
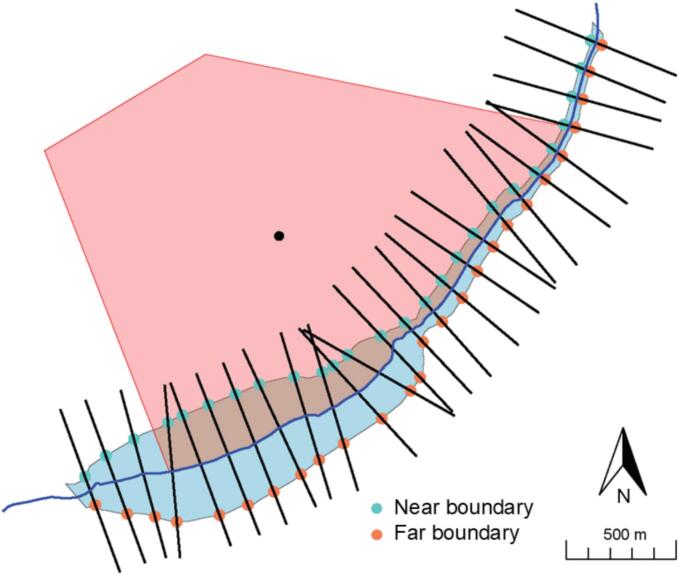
Schematic showing classification of near and far river boundaries. The red polygon is the village buffer, the blue area is the adjacent river section, and the black dot marks the buffer centroid. Transects (black lines) were drawn across the river to identify point-pairs on opposite banks, enabling classification of near and far boundaries, shown as turquoise and orange dots.

To measure river-cutting, we extracted the river section adjacent to each village buffer and created a centerline for each year. Points were sampled every 100 m along the centerline, and the slope between adjacent points were calculated. Perpendicular transects were then constructed at each sample point, extending across the river from the village center. The transects crossed both the near and far boundaries of the river—where the near boundary is the edge that can be reached without crossing the river, and the far boundary requires crossing the river (Fig. 4).

For each transect, the points where it intersected both river boundaries were recorded, creating a set of points that traced the river’s edges. Transects intersecting the river at more than two points were excluded to avoid capturing small kinks or branches. The village buffer’s bounding box was divided using a small buffer around the river centerline, and the section containing the village point was identified to classify which boundary points belonged to the near boundary. Distances from the village point to each near boundary point were then calculated, and the average of these distances represented the distance of the near river boundary from the village. This process was repeated for each year from 2016 to 2023 and for each village in the study sample.

#### 2.5.4 Topographical and environmental factors affecting village vulnerability and river morphology

We computed the “distance to sugar mill” as the Euclidean distance from each village center to the nearest of the three sugar mills in the region, serving as a proxy for village-level incentives to engage in sugarcane cultivation. Limited market access due to poor transportation infrastructure underscores the importance of proximity to processing facilities (Dhakal et al., 2016, Neupane et al., 2017, Pokharel et al., 2019). We also included the Euclidean distance to the nearest river and its position within the river system to assess flood risk and village vulnerability (Pandeya et al., 2021). We measured the distance to the nearest flood mitigation structure—typically small-scale, village-specific interventions like gabion walls or bamboo barriers—which serve as proxies for vulnerability to river-cutting and soil erosion (ICIMOD, 2013).

We generated variables to capture key topographic and hydrological conditions influencing soil erosion and river shifts. Flood risk was assessed at the village buffer level using a GIS-based hazard map from hydrological models of the Mohana and Macheli watersheds, with risk levels ranked from 0 (low) to 5 (high). We also calculated the average slope and elevation within 100 m of river sections adjacent to each village, reflecting terrain features that affect land use and flood vulnerability (Sugianto et al., 2022). River sinuosity was included to reflect geomorphological processes affecting land stability and river morphology (Frasson et al., 2019). In highly sinuous rivers, uneven water flow leads to enhanced erosion along outer bends and sediment deposition along inner bends, driving landscape change (Hooke, 2013). To quantify sinuosity, we delineated the river centerline next to each village and calculated the ratio of the river’s actual path length to the straight-line distance between its endpoints, generating an index that captures the degree of meandering and its potential effects on land use and river morphology.

We excluded coarse-resolution environmental and climate variables due to limited spatial variability across nearby villages and the scarcity of weather data, respectively. However, our DID model accounts for such factors through village fixed effects, controlling for time-invariant characteristics, and year fixed effects, addressing region-wide temporal shocks (Wooldridge, 2010).

### 2.6 Estimating causal effects

#### 2.6.1 Optimal full matching to address selection bias

To address selection bias from the non-random assignment of villages in the M−RED program—which prioritized communities with economic potential for sugarcane and vulnerability to river hazards—we employed OFM as direct comparisons between treated and untreated villages unreliable due to potential confounding factors (Dehejia and Wahba, 2002, Heckman et al., 1997).

Matching methods, such as nearest neighbor, caliper, and kernel matching, are commonly used to pair treated and control units based on their propensity scores—estimated treatment probabilities—to approximate random assignment and reduce confounding (Rosenbaum and Rubin, 1983, Stuart, 2010). However, our study’s small sample size—127 villages observed over eight years (N = 127, T = 8)—posed challenges for traditional matching methods, which often result in data loss and reduced statistical power (Ho et al., 2007). OFM overcomes these limitations by allowing flexible matching ratios (1:k or k:1) within subclasses, minimizing differences in propensity scores while retaining all observations (Hansen, 2004). It uses a network flow algorithm to find optimal match configurations, improving covariate balance and reducing within-subclass variation (Hansen, 2004, Hansen and Klopfer, 2006). Unlike “greedy” methods like nearest neighbor matching—which may yield suboptimal results—OFM achieves globally optimal matches by evaluating the full dataset (Rosenbaum, 2002). This makes OFM especially valuable in small samples, where retaining all units is critical for maintaining statistical power and estimating unbiased treatment effects (Gu and Rosenbaum, 1993). Given the potential for data loss in small samples, the advantages of OFM were crucial for maintaining the validity and precision of our analysis.

A key challenge was the lack of survey data on village characteristics typically used in matching models. To overcome this, we relied on remotely sensed variables as proxies for estimating propensity scores (Table 2). For instance, we included each village’s distance to flood mitigation infrastructure and its flood risk level—factors likely influencing both treatment probability and outcomes. Including these variables helped ensure treated and comparison villages were similarly exposed to flood risk, reducing confounding.

To construct a valid comparison group, we followed a two-step process. First, we estimated village-level propensity scores using a Probit model and identified nearby untreated villages from the UN OCHA settlement layer as potential matches. The model incorporated 2016 (pre-treatment) values of covariates derived from remote sensing, selected based on contextual relevance rather than statistical significance (Caliendo and Kopeinig, 2008). For example, the slope near the river influenced both the likelihood of treatment assignment and outcomes such as soil erosion. In the second step, we applied the OFM procedure to construct the final matched sample.

#### 2.6.2 Performing difference-in-differences analysis

While matching approaches can help ensure treated and comparison groups are balanced on observable characteristics, they cannot account for potential confounding factors that differ across these groups but are not observed or included in the analysis (Angrist & Pischke, 2009). To address this limitation, we utilize DID approaches that (a) control for unobserved factors that may differ across groups but that change only relatively slowly over time, such as many geographic conditions, and (b) leverage the staggered roll-out of interventions such as M−RED to further adjust for potentially confounding factors. Our approach uses the panel data structure in which each of the sample villages is observed for 8 years, and in which two-way fixed effects at both the village- and year-levels adjust for unobserved factors that are common to each village and those that are common to all villages in each year. That is, village fixed effects control for time-invariant characteristics specific to each village (e.g., soil type, elevation), while year fixed effects account for time-specific shocks that affect all villages simultaneously (e.g., regional climate conditions or macroeconomic changes faced by all villages in any year).

In addition to these fixed effects, we leverage the time variation across the treatment status of each M−RED village. As shown in Table 1 above, the overall M−RED program staggered in its roll-out, reaching a growing number of villages over our sample period. We use the time-varying treatment status of each village to further narrow our comparisons to those between already-treated villages and not-yet-treated villages, using the pool of never-treated comparison villages to primarily account for unobserved factors that are common to all locations each year. Our regression specification for our analysis is as follows:

$$y_{\mathrm{vt}}=\alpha +\beta *Treated_{\mathrm{vt}}+\delta_{v}+\delta_{t}+\varepsilon_{\mathrm{vt}}$$

where *y_vt_* refers to the outcome of interest for village, *v,* in year, *t*. *Treated_vt_* is a binary variable indicating whether village *v* was treated in year *t*. *δ_v_* and *δ_t_* represent village and year fixed effects, respectively. *β* is our coefficient of interest and estimate of treatment effects. The regression is weighted by the weights derived from OFM, which serve as inverse probability weights.

Recent work has shown that, when treatment effects are time-varying (such as when treatment leads to accumulating benefits over time), such DID models can be biased (Callaway and Sant’Anna, 2021). We thus use the method proposed by Callaway and Sant’Anna (2021) that excludes “forbidden comparisons” from the analysis (those between newly treated and already treated units).

#### 2.6.3 Robustness checks for validating causal models

In any quasi-experimental setting, violations of parallel trends, unobserved confounders, or model dependence can bias results if left unaddressed (Angrist & Pischke, 2009). Therefore, we implemented three key robustness checks to assess the sensitivity of estimated treatment effects to alternative specifications, assumptions, and potential bias (Athey & Imbens, 2017). First, we conducted a sensitivity analysis by progressively adding covariates to the probit model for generating propensity scores. We begin with the variable exhibiting the greatest imbalance, measured by standardized mean differences between the treatment and comparison groups, and sequentially add 13 covariates, concluding with the variable exhibiting the least imbalance. This stepwise approach allowed us to assess whether treatment effects remained stable as additional controls were introduced or if specific covariates drove the results. The degree of stability of estimated treatment effects in this test indicates whether they stem from model specification or reflect a true causal effect (Angrist & Pischke, 2009). Second, we accounted for potential pre-treatment differences in trends between treated and comparison villages due to river-specific environment and economic characteristics (Towolawi et al., 2024). If such differences exist and are unaccounted for, they could violate the parallel trends assumption and bias our estimates (Angrist & Pischke, 2009). By incorporating linear time trends for each river system, we controlled these systematic, longer-term trends, ensuring that the observed treatment effects are not a result of different trajectories (Athey & Imbens, 2017). Finally, we conducted a placebo test by randomly reassigning treatment cohort membership across the entire sample and re-estimating treatment effects. If spurious correlations had driven our estimates, we would observe similar treatment effects even with artificial treatment timing. However, if the M−RED program truly caused the observed effects, the estimated treatment effects in the placebo test would be statistically indistinguishable from zero (Angrist & Pischke, 2009). Together, these robustness checks strengthen our causal claims by addressing key identification threats, ensuring that our estimated treatment effects reflect true treatment effects rather than artifacts of model specification or selection bias.

## 3 Results

### 3.1 Effectiveness of OFM in enhancing comparability between treated and comparison groups

The OFM procedure reduced initial differences between treatment and comparison groups for 7 of the 12 variables in the Probit model, thereby enhancing the comparability of the comparison group (Table 3). For instance, the distance to the nearest sugar mill was significantly reduced from 6 km (*p* = 0.001) to 3 km (*p* > 0.05) with marginal significance after OFM weighting, indicating effective balancing. However, the mean elevation difference increased between groups after weighing (*p* < 0.05). Among the four strongest predictors of treatment—*distance to sugar mill*, *distance to river*, *mean elevation*, and *baseline sugarcane levels*—OFM was effective in reducing differences in the first three predictors of treatment. Similar results were obtained for the spillover sample, where initial differences were reduced across six variables (Table 4). Significant differences (*p* < 0.01) between the contaminated and pure comparisons, found only for *distance to sugar mill* and *mean slope*, were nullified after applying OFM weights. After weighing, *distance to river* and baseline sugarcane levels (i.e., percentage of land planted with sugarcane) become marginally significant (*p* > 0.05) (Table 4). These results demonstrate that OFM effectively controls confounding factors by pairing similar treatment and comparison units together, thereby improving their comparability and reducing selection bias.

**Table 3:** Group-level averages of Probit model variables for treatment and comparison villages, shown with and without Optimal Full Matching (OFM) weights. “Unweighted” excludes OFM weights, while “Weighted” includes them. Standard errors (S.E.) are in parentheses; “Observations” indicates the number of villages analyzed.

Variable	Unweighted means			Weighted means		
	Comparison	Treatment	Difference	Comparison	Treatment	Difference
Distance to sugar mill (m)	15537.1	9675.44	-5861.73***	12,714.54	9,675.44	-3,039.10*
	(6,547.75)	(5471.32)	(0.00)	(7,176.44)	(5,471.32)	(0.05)
Distance to river (m)	732.22	650.92	-81.30	523.26	650.92	127.67
	(478.53)	(441.15)	(0.47)	(367.28)	(441.15)	(0.21)
Distance to nearest flood infrastructure (m)	1371.60	1580.02	208.42	1,251.08	1,580.02	328.94
	(1,583.64)	(2174.81)	(0.66)	(1,431.73)	(2,174.81)	(0.48)
Flood risk level	1.92	2.51	0.59	2.12	2.51	0.39
	(1.93)	(1.83)	(0.21)	(1.18)	(1.83)	(0.31)
Population count (indv.)	2042.55	2061.72	19.17	2,001.40	2,061.72	60.32
	(2752.77)	(1789.95)	(0.97)	(1,978.84)	(1,789.95)	(0.90)
Built-up area (m^2^)	60599.90	52780.39	-7819.51	65,506.15	52,780.39	-12725.76
	(50184.50)	(35094.24)	(0.46)	(38,773.56)	(35,094.24)	(0.16)
Mean slope (degrees)	3.22	2.52	-0.70***	2.98	2.52	-0.46**
	(1.07)	(0.76)	(0.00)	(0.67)	(0.76)	(0.01)
Mean elevation (m)	182.77	172.94	-9.829*	183.95	172.94	-11.01**
	(27.80)	(16.49)	(0.078)	(23.04)	(16.49)	(0.03)
Average baseline sinuosity	1.29	1.25	-0.042	1.29	1.25	-0.04
	(0.24)	(0.31)	(0.533)	(0.14)	(0.31)	(0.56)
Baseline pct. land planted with sugarcane	0.16	0.26	0.10**	0.16	0.26	0.10**
	(0.13)	(0.20)	(0.02)	(0.13)	(0.20)	(0.02)
Baseline pct. land affected bysoil erosion	0.21	0.25	0.05	0.21	0.25	0.05
	(0.16)	(0.19)	(0.28)	(0.16)	(0.19)	(0.28)
Baseline average distance to near boundary (m)	1,138.09	1,234.36	96.28	1,138.09	1,234.36	96.28
	(512.98)	(465.54)	(0.42)	(512.98)	(465.54)	(0.42)
Observations	31	36	67	31	36	67

Standard errors in parentheses.

* p<0.1 ** p<0.05 *** p<0.01.

**Table 4:** Group-level averages of Probit model variables for the spillover sample (contaminated vs. pure comparisons), shown with and without Optimal Full Matching (OFM) weights. “Unweighted” excludes weights, while “Weighted” includes them. Mean differences, standard errors (S.E.), and number of villages (Observations) are also reported.

Variable	Unweighted means			Weighted means		
	Contaminated	Pure	Difference	Contaminated	Pure	Difference
Distance to sugar mill (m)	10,136.457	15,537.168	5,400.711***	10,136.457	10,869.553	733.096
	(4,993.688)	(6,547.745)	(0.001)	(4,993.688)	(6,742.961)	(0.653)
Distance to river (m)	624.903	732.219	107.316	624.903	994.431	369.528***
	(430.766)	(478.529)	(0.387)	(430.766)	(536.203)	(0.007)
Distance to nearest flood infrastructure (m)	1,965.439	1,371.600	-593.839	1,965.439	1,303.051	-662.389
	(2,053.486)	(1,583.634)	(0.227)	(2,053.486)	(1,449.415)	(0.164)
Flood risk level	2.406	1.920	-0.486	2.406	2.091	-0.315
	(2.058)	(1.925)	(0.366)	(2.058)	(1.454)	(0.506)
Population count	1,767.400	2,042.548	275.148	1,767.400	2,324.448	557.048
	(2,740.102)	(2,752.765)	(0.711)	(2,740.102)	(2,139.394)	(0.397)
Built-up area (m^2^)	43,403.602	60,599.902	17,196.303	43,403.602	59,309.941	15,906.340
	(50,832.051)	(50,184.500)	(0.210)	(50,832.051)	(32,030.322)	(0.159)
Mean slope	2.467	3.222	0.755***	2.467	2.760	0.294
	(0.690)	(1.070)	(0.004)	(0.690)	(0.863)	(0.173)
Mean elevation	174.989	182.766	7.777	174.989	179.719	4.730
	(19.070)	(27.800)	(0.239)	(19.070)	(21.408)	(0.392)
Average baseline sinuosity	1.256	1.293	0.037	1.256	1.276	0.020
	(0.294)	(0.237)	(0.605)	(0.294)	(0.178)	(0.751)
Baseline pct. land planted with sugarcane	0.178	0.159	-0.019	0.178	0.251	0.073*
	(0.097)	(0.133)	(0.546)	(0.097)	(0.177)	(0.070)
Baseline pct. land affected by soil erosion	0.238	0.206	-0.032	0.238	0.186	-0.052
	(0.211)	(0.159)	(0.522)	(0.211)	(0.158)	(0.300)
Baseline average distance to near boundary (m)	1,154.758	1,138.089	-16.669	1,154.758	1,267.328	112.570
	(377.628)	(512.977)	(0.893)	(377.628)	(580.132)	(0.406)
Observations	25	31	56	25	31	56

Standard errors in parentheses.

* p<0.1 ** p<0.05 *** p<0.01.

### 3.2 Effect of M−RED intervention on sugarcane adoption on riverbank degradation

**Fig. 5:**
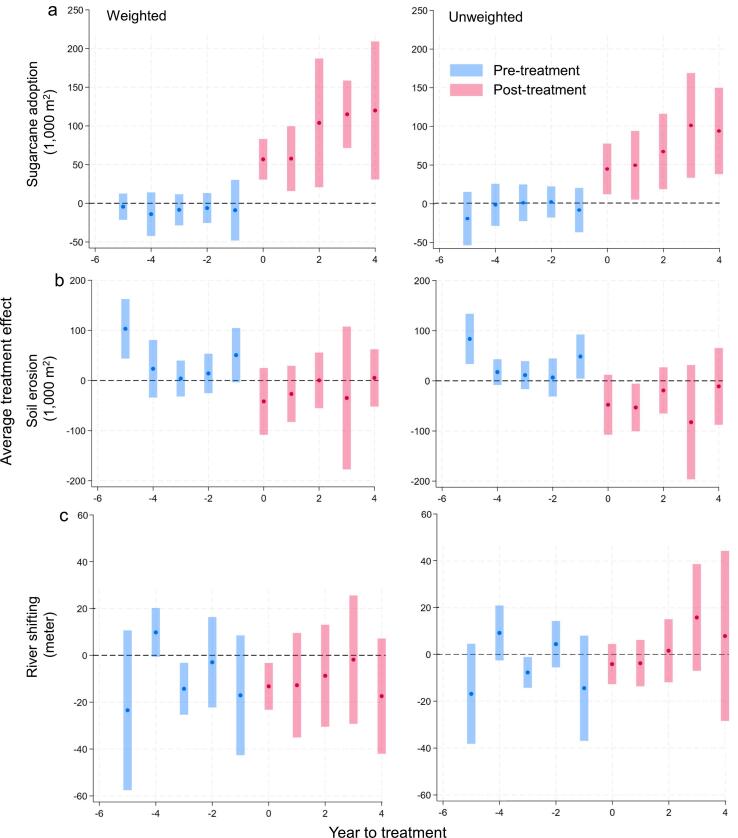
Effects of the M-RED program on (a) sugarcane adoption, (b) soil erosion, and (c) river shifting over time. Each panel shows weighted and unweighted results, with blue (pre-treatment) and red (post-treatment) points indicating average outcome differences between treated and comparison groups. Shaded bands denote 95% confidence intervals.

Incorporating matching weights from OFM into the DID model provided a robust framework for estimating the causal impacts of sugarcane adoption while effectively controlling confounding factors and selection bias in the evaluation of the M−RED intervention. Sugarcane adoption in treated communities increased non-linearly over four years following the M−RED intervention (Fig. 5a, Weighted), contrasting with the stable pre-treatment trend. This consistent growth, observed regardless of OFM weighting (Fig. 5a, Unweighted), highlights the program’s effectiveness. Before the intervention (T-5 through T-1), the differences in sugarcane adoption between treatment and comparison groups were small and statistically insignificant (|t| < 1.20) in the weighted sample (Table 5, column 1). Post-intervention (T-0), treated communities exhibited a significant increase of 56,830 m2 in sugarcane adoption (|t| = 4.25; *p* = 001), which persisted throughout the post-treatment period (T-0 to T-4), and by the fourth year, this difference raised to 120,000 m^2^, making a 110 % increase from T-0 (|t| = 2.64). These trends were consistently observed in both the weighted and unweighted samples. In contrast, the effects on soil erosion and river shifting were more modest, with inconsistent statistical significance across the pre- and post-treatment period (Fig. 5 b-c, Weighted and Unweighted). For soil erosion (Table 5, column 2), differences between treatment and comparison groups fluctuated, with some reaching statistical significance (*p* < 0.01) and others remaining insignificant (*p* > 0.1). Post-treatment, differences in soil erosion were consistently small and insignificant (*p* > 0.1). Similarly, river shifting (Table 5, column 3) showed a significant decrease of 0.01 m in the first post-treatment year (|t| = 2.59), but this effect did not persist (*p* > 0.10). These results suggest strong positive impacts for sugarcane adoption, with limited effects on environmental variables.

**Table 5:** Coefficients from an event-study using the Callaway and Sant’Anna (2021) DID methodology, covering five pre-treatment and four post-treatment periods. T-statistics are in parentheses and all models include village and year fixed effects (FE).

Period	Sugarcane adoption (1,000 m^2^)		Soil erosion (1,000 m^2^)		River shifting (meters)	
	Weighted	Unweighted	Weighted	Unweighted	Weighted	Unweighted
	(1)	(2)	(3)	(4)	(5)	(6)
T-5	-4.386	-19.93	103.6***	83.69***	-0.0235	-0.0169
(t-statistic)	(-0.51)	(-1.13)	(3.27)	(3.27)	(-1.35)	(-1.54)
T-4	-14.08	-2.115	23.12	17.49	0.00978*	0.00922
	(-0.98)	(-0.15)	(0.76)	(1.33)	(1.84)	(1.54)
T-3	-8.474	0.414	3.798	11.43	-0.0143**	-0.00775**
	(-0.83)	(0.03)	(0.26)	(0.80)	(-2.52)	(-2.31)
T-2	-6.137	1.502	11.67	6.677	-0.00298	0.00441
	(-0.62)	(0.15)	(0.49)	(0.34)	(-0.30)	(0.87)
T-1	-8.931	-9.024	50.54*	48.54**	-0.0171	-0.0145
	(-0.45)	(-0.62)	(1.74)	(2.16)	(-1.31)	(-1.26)
T+0	56.83***	44.24***	-41.08	-47.72	-0.0132***	-0.00413
	(4.25)	(2.63)	(-1.28)	(-1.56)	(-2.59)	(-0.95)
T+1	57.74***	49.01**	-31.16	-53.07**	-0.0128	-0.00375
	(2.70)	(2.16)	(-1.14)	(-2.19)	(-1.12)	(-0.74)
T+2	103.9**	66.79***	1.424	-19.09	-0.00874	0.00162
	(2.45)	(2.69)	(0.06)	(-0.81)	(-0.79)	(0.24)
T+3	115.0***	100.6***	-37.96	-82.47	-0.00184	0.0158
	(5.17)	(2.91)	(-0.55)	(-1.42)	(-0.13)	(1.36)
T+4	120.0***	93.34***	12.18	-10.95	-0.0174	0.00791
	(2.64)	(3.27)	(0.40)	(-0.28)	(-1.39)	(0.43)
N	67	67	67	67	67	67
Village FE	Yes		Yes		Yes	
Year FE	Yes		Yes		Yes	

t-statistics in parentheses.

* p<0.1 ** p<0.05 *** p<0.01.

### 3.3 Spillover effect of sugarcane adoption on comparison communities

**Fig. 6:**
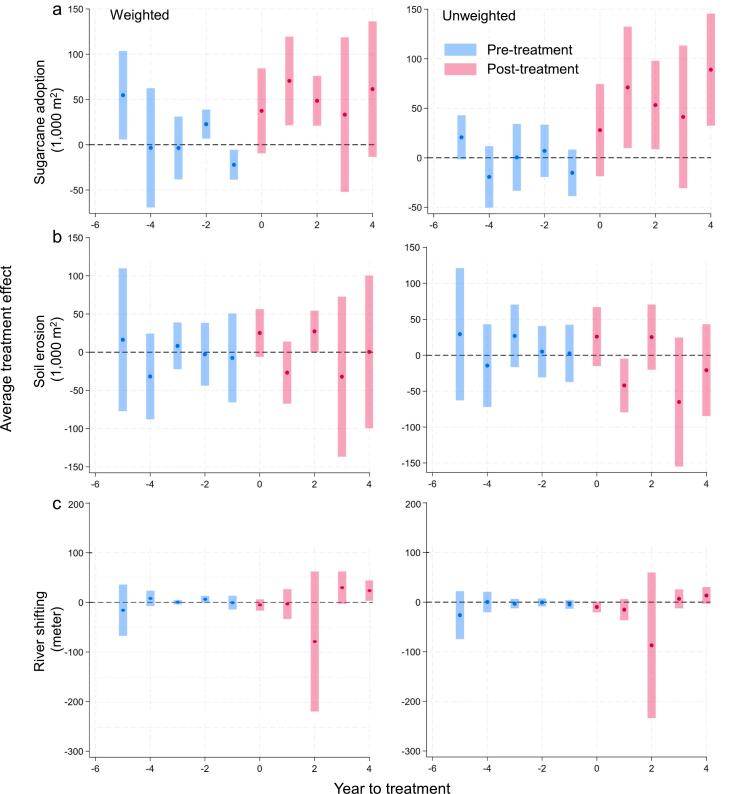
Impacts of the M-RED program on (a) sugarcane adoption, (b) soil erosion, and (c) river shifting in adjacent spillover communities. Panels show weighted and unweighted results; blue and red points indicate average differences between treatment and comparison groups pre- and post-treatment. Shaded bands denote 95% confidence intervals.

Sugarcane adoption in contaminated comparison communities, i.e., spillover communities, showed significant increases (*p* < 0.01) in the second- and third-years post-treatment. Despite positive coefficients throughout the post-treatment period, the lack of significance suggested weaker adoption compared to directly treated communities (Fig. 6). Pre-treatment differences in sugarcane cultivation between groups were larger than in the main sample, i.e., the sample with villages treated under M−RED, with some pre-treatment coefficients significant in the weighted sample but not in the unweighted one (Fig. 6; Table 6, columns 1 and 2). No discernible pre-treatment trends were observed, indicating no differential trends between the contaminated and pure comparisons before the intervention. In the first year of treatment, sugarcane adoption increased in spillover communities, with significant growth in years 1 and 2 for the weighted sample (*p* < 0.01) (Table 6, column 1) and years 1, 2, and 4 in the unweighted sample (*p* < 0.05) (Table 6, column 2). Although sugarcane adoption continued to grow post-treatment, the impact on soil erosion and river shifting was inconsistent. Soil erosion coefficients remained statistically insignificant throughout the post-treatment period (Fig. 6b; Table 6, column 3 and 4). For river shifting, treatment effects showed a favorable upward trend (Fig. 6c), though the coefficients were insignificant, except in year 4 for the weighted sample (Table 6, column 5 and 6).

**Table 6:** Event-study coefficients from the Callaway and Sant’Anna (2021) DID model for spillover communities, covering 5 pre- and 4 post-treatment periods. The t-statistics are in parentheses. All specifications include fixed effects (FE) at the village and year level.

Period	Sugarcane adoption (1,000 m^2^)		Soil erosion (1,000 m^2^)		River shifting (meters)	
	Weighted	Unweighted	Weighted	Unweighted	Weighted	Unweighted
	(1)	(2)	(3)	(4)	(5)	(6)
T-5	54.75**	20.64*	16.30	29.36	-15.88	-26.23
	(2.20)	(1.82)	(0.34)	(0.62)	(-0.61)	(-1.06)
T-4	-3.379	-19.33	-31.73	-14.42	7.706	0.342
	(-0.10)	(-1.22)	(-1.11)	(-0.49)	(0.98)	(0.03)
T-3	-3.557	0.358	8.318	27.00	0.109	-3.012
	(-0.20)	(0.02)	(0.53)	(1.21)	(0.05)	(-0.63)
T-2	22.75***	6.936	-2.747	5.021	6.022*	-0.575
	(2.78)	(0.51)	(-0.13)	(0.28)	(1.74)	(-0.14)
T-1	-22.11***	-15.25	-7.446	2.580	-0.762	-4.657
	(-2.62)	(-1.27)	(-0.25)	(0.13)	(-0.11)	(-1.04)
T-0	37.46	27.87	25.17	26.03	-5.271	-9.707*
	(1.56)	(1.17)	(1.58)	(1.25)	(-0.91)	(-1.77)
T+1	70.54***	71.04**	-26.75	-42.08**	-3.493	-15.19
	(2.82)	(2.27)	(-1.29)	(-2.21)	(-0.23)	(-1.40)
T+2	48.57***	53.19**	27.26**	25.27	-78.38	-86.93
	(3.45)	(2.33)	(1.96)	(1.09)	(-1.10)	(-1.16)
T+3	33.28	41.23	-32.02	-65.04	29.31*	6.734
	(0.76)	(1.12)	(-0.60)	(-1.42)	(1.77)	(0.69)
T+4	61.45	88.94***	0.437	-20.76	23.30**	13.42
	(1.61)	(3.08)	(0.01)	(-0.64)	(2.24)	(1.56)
N	56	56	56	56	56	56
Village FE	Yes		Yes		Yes	
Year FE	Yes		Yes		Yes	

t-statistics in parentheses.

* p<0.1 ** p<0.05 *** p<0.01.

## 4 Robustness checks for causal inference

**Fig. 7:**
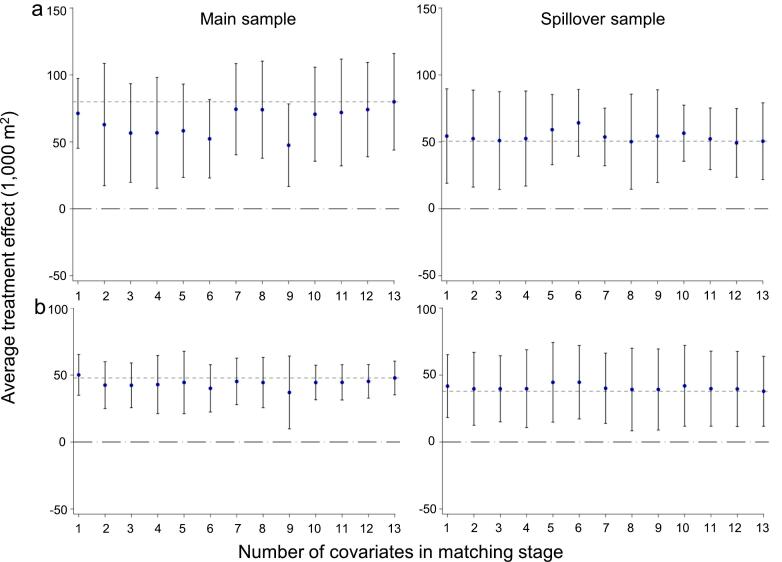
Average treatment effects on sugarcane adoption in (a) main and (b) spillover samples, sequentially adding covariates to the propensity score model by decreasing imbalance. Black points show outcome differences with 95% confidence intervals; the dashed gray line marks the ATE using all 13 covariates.

**Fig. 8:**
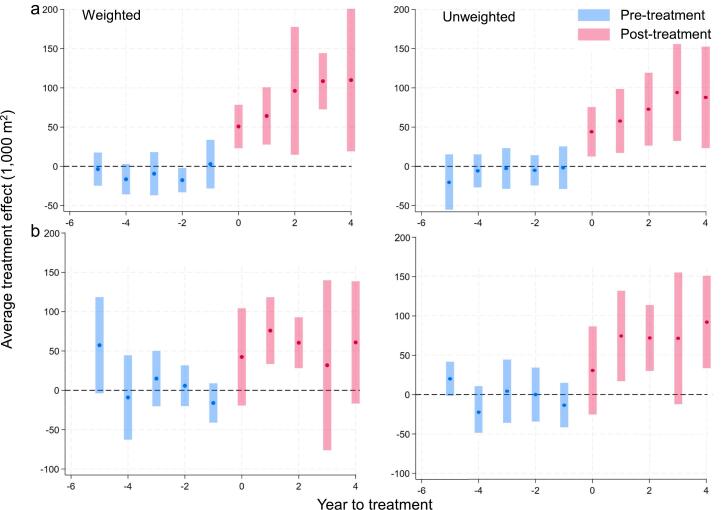
The effect of the M-RED program on sugarcane adoption over time in (a) main and (b) spillover samples, accounting for river-specific time trends. Each panel shows weighted and unweighted results; blue and red points indicate pre- and post-treatment differences with 95% confidence bands.

**Fig. 9:**
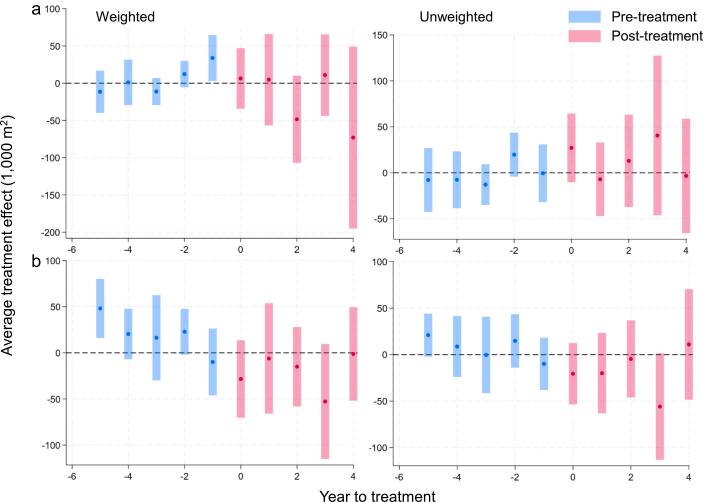
Placebo test for sugarcane adoption in (a) main and (b) spillover samples using randomly assigned treatment dates. Panels show weighted and unweighted results; blue and red points indicate pre- and post-treatment differences with 95% confidence bands.

The impact of the M−RED intervention on sugarcane adoption remained consistent across three key robustness checks. Both the Callaway and Sant’Anna (2021) and two-way fixed effects (FE) estimators produced stable average treatment effects (ATEs) across all 13 model specifications (Fig. 7). The final ATE, incorporating all variables, remained statistically significant and fell within the 95 % confidence interval of all but one Callaway-Sant’Anna estimate in the main sample, and within all confidence intervals in the spillover sample, demonstrating robustness to variable selection and effective bias control. Similarly, river-specific time trends did not alter the direction or magnitude of treatment effects, confirming that pre-existing temporal patterns did not confound the estimates. These results closely matched those from the main DID specification in both samples (Fig. 8; Table A1; Table A2), with all estimates falling within the respective confidence intervals (Fig. 5; Fig. 6; Fig. 8). Moreover, a placebo test with randomly assigned treatment dates found no significant effects (Fig. 9), reinforcing the causal interpretation of our findings. Collectively, these tests confirmed that the estimated treatment effects were not artifacts of model specification, temporal confounders, or random variation, strengthening their internal validity.

## 5 Discussion

Our study evaluated the Mercy Corps’ M−RED program, a low-cost intervention promoting sugarcane cultivation in flood-prone communities of western Nepal. We aimed to assess whether sugarcane could serve as a sustainable agricultural practice with economic and environmental benefits. Findings show adoption in treated villages rose by 23 % in the first year and nearly 50 % after four years. Spillover effects were also observed: nearby non-treated villages experienced a 43 % increase by year two and 30 % by year three, though later-year impacts were not statistically significant. These results support the potential of combining economic incentives with sustainable farming to boost adoption in vulnerable regions (Piñeiro et al., 2020). However, expected environmental impacts—such as reductions in soil erosion and riverbank cutting (Cheng et al., 2022) —were not statistically significant, suggesting such benefits may need longer to materialize or require more targeted interventions. The disconnect highlights the importance of refining both the scale and design of similar future efforts.

Our findings of no significant impact on river cutting and soil erosion align with prior research. For instance, Cheng et al. (2022) observed erosion control benefits from sugarcane only when combined with grass cover, a known erosion suppressant (Gholami and Khaleghi, 2013, Kafle and Balla, 2008, Mondal and Patel, 2020). Similarly, studies in Brazil and Australia have shown that sugarcane’s environmental benefits vary with local conditions (Prove et al., 1995, Sparovek and Schnug, 2001). This may also stem from converting forests to sugarcane fields, which, while expanding cultivated land, likely reduced soil organic matter and increased susceptibility to riverbank erosion. Abdalla et al. (2019) and Li et al (2021) emphasize the importance of organic carbon and vegetation cover for erosion control, particularly on steep slopes. Sugarcane may also have been planted in already degraded or marginal areas, limiting its effectiveness. The Terai’s unique hydrology—marked by high-flow variability and seasonal floods—may further explain the absence of measurable environmental benefits. These findings point to the need for more targeted land management strategies and context-specific assessments of sugarcane’s ecological impact.

Similarly, our failure to detect significant effects on river cutting aligns with previous studies. Unlike surface soil erosion, river cutting involves the lateral migration of channels—a slower process that often requires longer observation periods to assess impact (Brookes, 1994). The four-year post-intervention study period may have been insufficient to observe meaningful changes from sugarcane cultivation, which typically needs more time to establish vegetation capable of withstanding peak flows (Bariteau et al., 2013). For example, Huang et al. (2019) utilized a 10-year study period to examine the stabilizing effects of Vetiver grass. Moreover, the effectiveness of bioengineering measures like sugarcane planting is often site-specific, influenced by factors such as flow velocity and erosion severity (Rauch et al., 2022, Mondal and Patel, 2020). In western Nepal’s Terai, high flow variability and intense seasonal floods may have overwhelmed the limited gains from increased sugarcane coverage (Olabisi, 2012). Additionally, replacing native vegetation—which may offer stronger riverbank protection (Lann et al., 2024, Zhu et al., 2018)—could have reduced overall stabilization benefits. These findings highlight the complexity of addressing riverbank erosion through bioengineering in dynamic environments.

This study contributes to the theoretical understanding of bio-engineering interventions for environmental resilience, particularly in the context of sustainable agriculture. While many studies have contributed to this topic, their focus on small-scale, region-specific interventions with limited temporal variability limits the generalizability of their findings. For example, Cheng et al. (2022) conducted a 2-year analysis of a localized soil stabilization project involving sugarcane, highlighting its effectiveness. Similarly, Gholami and Khaleghi (2013) focus on a 3250-meter section of the Haraz River in Iran over just two seasons to analyze the suitability of vegetation as a bio-engineering measure for erosion control. Some studies use experimental setups but are limited by small spatial scales and short timeframes, such as Vasquez-Mendez et al. (2010), who monitor just four plots for six months, or Oku et. al. (2014), who focus on a single farmer’s field in Nigeria. In contrast, our research contributes novel insights by incorporating significant temporal variability across a large, regionally diverse area and applying advanced causal identification techniques to strengthen the robustness of our findings. Few studies have rigorously evaluated the effectiveness of vegetation-based bio-engineering measures using causal identification techniques, and traditional site-based studies often overlook key methodological challenges, such as selection bias and confounding factors, which can lead to biased assessments of bio-engineering efficacy (Cheng et al., 2022, Mondal and Patel, 2020).

We applied a rigorous causal identification strategy by combining OFM with a DID design to estimate the effect of sugarcane adoption on riverbank stability, while minimizing confounding bias. This approach allowed us to construct a credible counterfactual by retrospectively identifying comparable untreated villages and controlling pre-existing differences. However, several limitations remain. First, the analysis assumes sugarcane adoption is the primary driver of observed changes in riverbank outcomes. Despite controlling for observable factors, unmeasured influences—such as broader land-use practices, hydrological shifts, or governance differences—may still confound results (Caliendo and Kopeinig, 2008). Spatial analysis confirmed no systematic treatment allocation, and interpreters were blinded to treatment status, reducing measurement bias. Manual digitization errors were likely random and preferable to automated methods, which may misclassify complex landscape features (Huang et al., 2021). Second, the analysis was constrained by sample size and data limitations. With only about 70 villages included and no pre-treatment data for those treated in 2016, statistical power to detect subtle effects was limited. As such, null results may reflect low power rather than true absence of impact (Singh and Masuku, 2014). Future studies with broader coverage and longer time frames are needed to more definitively assess the environmental benefits of sugarcane-based bioengineering interventions.

Remote sensing provided a strong foundation for monitoring land cover change and informing causal analysis in our study, enabling accurate regional mapping of sugarcane fields and relevant land characteristics, consistent with findings by Jain (2020) and Magliocca et al. (2023). Random forest and clustering algorithms performed well in mapping sugarcane with high accuracy, aligning with findings of Luciano et al. (2019). However, the absence of annual ground-truth crop data during the M−RED period, as highlighted by Stehman and Foody (2019), likely introduced omission and commission errors, an issue that, if addressed, could enhance the robustness of future GIEs. Likewise, incorporating the rate of change in forested areas, which previously safeguarded riverbanks, could further enhance the GIE outcomes. While soil erosion and river cutting estimates derived from visual interpretation were not statistically significant, they offered valuable insights into riverbank dynamics, complementing bioengineering measures recommended by Marteau et al. (2018). Likewise, Elevation and river sinuosity data from high-resolution digital elevation models also aided in understanding erosion patterns (Chalise et al., 2019). Although measurements derived from remote sensing data contributed substantially to our GIE, it may have missed fine-scale variations critical to understanding sugarcane’s impact on stabilization. Manual digitization of erosion and river boundaries is subject to image quality and the experience of the interpreters (White, 2019). These challenges may have limited our ability to detect sugarcane’s full impact on river shifting and erosion. Future research would benefit from integrating field data—such as crop verification, soil testing, and riverbank surveys—and employing longer-term or experimental designs (e.g., randomized controlled trials) to more definitively assess the environmental impacts of sugarcane adoption.

These findings offer key insights into the M−RED program’s sugarcane initiative as a low-cost strategy for promoting sustainable agriculture in western Nepal. While the program significantly increased sugarcane cultivation in treated communities and triggered spillovers in nearby areas, expected environmental benefits—such as reduced soil erosion and river cutting—did not materialize. This suggests such outcomes may require longer timeframes, complementary vegetation types, or additional interventions to be realized. The study highlights the challenges of implementing bioengineering solutions in dynamic environments like the Terai, where hydrological and topographic conditions can affect intervention success. Our results point to the need for further research using larger samples, longer timelines, and context-specific strategies to better assess and improve the environmental effectiveness of such programs. Despite limited environmental effects, the study advances understanding of how economic incentives can drive sustainable practices. It also illustrates the value of the GIE framework for evaluating development programs in complex, data-scarce regions.

## 6 Conclusions

GIE enhances the evaluation of development program outcomes, enabling organizations to optimize investments for greater cost-effectiveness and impact while providing valuable insights for decision-makers, organizations, and researchers. Our GIE results offer strong evidence supporting the effectiveness of Mercy Corps’ M−RED intervention in promoting sugarcane adoption among vulnerable communities of western Nepal. The consistent increase in sugarcane cultivation over four years, both in treated and spillover communities, demonstrates the potential of low-cost interventions to encourage sustainable agriculture, especially when combined with economic incentives. The use of OFM within the DID framework, with data from remote sensing, successfully mitigated selection bias, refining causal impact estimates and confirming the positive effects on sugarcane adoption. However, while sugarcane adoption increased significantly, the expected environmental benefits, such as soil erosion control and riverbank stabilization, were not fully realized. The modest and inconsistent effects on these outcomes suggest that more targeted interventions or longer timeframes may be needed for substantial environmental changes. The spillover effects in non-treated communities further suggest that economic incentives can extend beyond directly treated areas. These findings highlight that while economic incentives can drive sustainable agricultural practices, environmental benefits may require additional interventions or time to manifest. The robustness of our results, confirmed by multiple sensitivity tests, including the Callaway and Sant’Anna (2021) estimator and placebo tests, strengthens the reliability of our conclusions. Future research should consider complementary interventions, such as integrating other vegetation types with sugarcane, and extended study duration to better capture long-term environmental impacts. This study provides valuable insights into the effectiveness of low-cost, scalable interventions for promoting sustainable agriculture in regions facing environmental degradation.

## CRediT authorship contribution statement

**Pratap Khattri:** Writing – review & editing, Writing – original draft, Visualization, Methodology, Formal analysis. **Rachel Sayers:** Writing – original draft, Supervision, Methodology, Formal analysis. **Kunwar K. Singh:** Writing – review & editing, Writing – original draft, Visualization, Supervision, Methodology, Funding acquisition, Formal analysis, Conceptualization. **Ryan Slapikas:** Formal analysis. **Chet Bahadur Tamang:** Formal analysis. **Dinee Tamang:** Formal analysis. **Brad Sagara:** Writing – original draft, Funding acquisition, Conceptualization. **Ariel BenYishay:** Writing – review & editing, Funding acquisition, Conceptualization.

## Declaration of competing interest

The authors declare that they have no known competing financial interests or personal relationships that could have appeared to influence the work reported in this paper.

## Figures and Tables

**Table A1:** Table shows event-study coefficients from the Callaway and Sant’Anna (2021) DID model with river-specific linear time trends. Estimates cover five pre-treatment and four post-treatment periods for main and spillover samples. T-statistics are in parentheses. All models include village and year fixed effects and river-specific trends.

Period	Main Sample		Spillover Sample	
	Weighted	Unweighted	Weighted	Unweighted
T-5	−3.550	−20.35	57.31*	19.99*
	(−0.33)	(−1.12)	(1.84)	(1.81)
T-4	−16.42*	−5.656	−9.071	–22.40
	(−1.67)	(−0.53)	(−0.33)	(−1.33)
T-3	−9.405	−2.682	14.93	4.226
	(−0.67)	(−0.20)	(0.83)	(0.21)
T-2	−17.62**	−5.067	5.843	−0.134
	(−2.23)	(−0.52)	(0.44)	(−0.01)
T-1	2.803	−1.784	−16.08	−13.49
	(0.18)	(−0.13)	(−1.26)	(−0.94)
T-0	50.80***	43.90***	42.41	30.50
	(3.59)	(2.73)	(1.34)	(1.07)
T + 1	64.20***	57.65***	75.90***	74.13**
	(3.44)	(2.78)	(3.51)	(2.53)
T + 2	96.23**	72.62***	60.47***	71.74***
	(2.32)	(3.07)	(3.67)	(3.37)
T + 3	108.5***	93.93***	31.85	71.20*
	(5.94)	(2.99)	(0.58)	(1.68)
T + 4	109.9**	87.67***	60.86	91.75***
	(2.37)	(2.66)	(1.54)	(3.07)
N	67	67	56	56
Village FE	Yes		Yes	
Year FE	Yes		Yes	
River-specific Linear Time-Trends	Yes		Yes	

t-statistics in parentheses.

* p < 0.1 ** p < 0.05 *** p < 0.01.

**Table A2:** Coefficients from an event-study analysis using the Callaway and Sant’Anna (2021) DID method with artificial treatment dates. It includes coefficients for 5 pre-treatment and 4 post-treatment periods for both main and spillover samples, with t-statistics in parentheses. All models account for village and year fixed effects.

Period	Main Sample		Spillover Sample	
	Weighted	Unweighted	Weighted	Unweighted
T-5	−11.48	−7.829	48.17***	20.98*
	(−0.79)	(−0.44)	(2.95)	(1.77)
T-4	1.113	−7.658	20.41	8.705
	(0.07)	(−0.48)	(1.46)	(0.52)
T-3	−11.18	−12.84	16.34	−0.345
	(−1.21)	(−1.13)	(0.69)	(−0.02)
T-2	12.23	19.74	22.84*	14.75
	(1.36)	(1.62)	(1.81)	(1.01)
T-1	33.85**	−0.530	−9.884	−9.929
	(2.16)	(−0.03)	(−0.53)	(−0.69)
T-0	6.392	27.07	−28.31	−20.64
	(0.31)	(1.42)	(−1.32)	(−1.23)
T + 1	4.822	−7.044	−6.094	−19.98
	(0.15)	(−0.34)	(−0.20)	(−0.91)
T + 2	−48.31	12.96	−14.99	−4.720
	(−1.62)	(0.51)	(−0.68)	(−0.22)
T + 3	10.91	40.61	−52.78*	−55.86*
	(0.39)	(0.92)	(−1.66)	(−1.91)
T + 4	−72.94	−3.395	−1.225	10.93
	(−1.17)	(−0.11)	(−0.05)	(0.36)
N	67	67	56	56
Village FE	Yes		Yes	
Year FE	Yes		Yes	

t-statistics in parentheses.

* p < 0.1 ** p < 0.05 *** p < 0.01.

## Data Availability

The data that has been used is confidential.
